# Neoadjuvante Therapie bei Hautkrebs: aktuelle Datenlage und Ausblicke

**DOI:** 10.1111/ddg.15968_g

**Published:** 2026-04-08

**Authors:** Lea Daniello, Johannes Kleemann, Bastian Schilling

**Affiliations:** ^1^ Klinik für Dermatologie Venerologie und Allergologie Universitätsmedizin Frankfurt Frankfurt am Main

**Keywords:** Hautkrebs, Melanom, Neoadjuvante Therapie, melanoma, neoadjuvant, skin cancer

## Abstract

Die Entwicklung von Immuncheckpoint‐Inhibitoren sowie zielgerichteten Therapien hat die Behandlung von Hauttumoren, vor allem des kutanen Plattenepithelkarzinoms, Melanoms und Merkelzellkarzinoms, grundlegend verändert. Neuste neoadjuvante Ansätze zeigen besonders bei lokal fortgeschrittenen Stadien nun praxisrelevante Ergebnisse.

Beim Plattenepithelkarzinom konnte die neoadjuvante PD‐1‐Blockade mit Cemiplimab in Studien ein gutes pathologisches Ansprechen erzielen. Zusätzlich weisen erste Daten auf ein geringeres Rezidivrisiko hin. Beim Melanom zeigen sich noch stärkere Fortschritte: Die neoadjuvante PD‐1‐Blockade, sowohl allein oder in Kombination mit einem CTLA‐4‐Blocker, reduziert signifikant das Rezidivrisiko. Sowohl die randomisierte Phase‐II‐Studie SWOG1801 als auch die randomisierte Phase‐III‐Studie NADINA zeigten die Überlegenheit des neoadjuvanten gegenüber einem rein adjuvanten Therapiekonzept. Die Ergebnisse der NADINA‐Studie zeigten, dass bei tiefem pathologischem Ansprechen auf die neoadjuvante Therapie eine Therapie‐Deeskalation durch Verzicht auf eine adjuvante Behandlung möglich ist. Auch zielgerichtete Therapien mit BRAF‐ und MEK‐Inhibitoren zeigen relevante Ansprechraten bei BRAF‐mutierten Melanomen. Beim Merkelzellkarzinom zeigt die neoadjuvante Gabe von PD‐1‐Inhibitoren wie Nivolumab eine hohe Ansprechrate sowie gute Überlebensdaten. Zusammenfassend unterstreichen die Daten das Potenzial der neoadjuvanten Therapie in der Behandlung lokal fortgeschrittener Hauttumoren mit Senkung von Rezidivrisiko und Mortalität sowie neuen Möglichkeiten der Therapiedeeskalationen.

## EINLEITUNG

Hautkrebs ist weltweit eine der häufigsten Krebserkrankungen und umfasst verschiedene Entitäten mit unterschiedlichen histologischen und molekularen Merkmalen. Die wichtigsten Formen sind keratinozytäre Hauttumoren (KH) und das Melanom. Zu den KH gehören das Plattenepithelkarzinom (cuSCC) und das Basalzellkarzinom, welche die Mehrheit aller Hauttumoren ausmachen.^1^ In den letzten Jahren mehren sich zudem Hinweise, dass zumindest ein Teil der Merkelzellkarzinome (MCC), welche eine seltenere Entität darstellen, ebenfalls keratinozytären Ursprungs sein könnten.^2^ Weitere seltene Hauttumoren sind Sarkome der Haut sowie B‐ und T‐Zell Lymphome der Haut.

In frühen Stadien können die meisten Hauttumoren chirurgisch kurativ behandelt werden. Insbesondere bei keratinozytären Tumoren spielt die Strahlentherapie sowohl in der primären als auch in der adjuvanten Therapie sowie in der fortgeschrittenen, inoperablen Situation eine Rolle. In fortgeschrittenen oder metastasierten Fällen werden primär zielgerichtete Therapien (TT) (nur beim Melanom) sowie Immuntherapien mit Immuncheckpoint‐Blockade (ICI) eingesetzt.^3^, ^4^, ^5^ Mit Einführung der ICI und TT hat sich das rezidivfreie Überleben (RFS) und das Gesamtüberleben (OS) bei MCC, cuSCC und Melanom deutlich verbessert. Zunehmend werden nun diese Systemtherapien auch neoadjuvant in Studien eingesetzt, wenngleich bisher oft nur in kleinen Patientengruppen. In den Jahren 2023 und 2024 wurden erstmals größere randomisierte Phase‐II‐ und Phase‐III‐Studien zur neoadjuvanten Therapie des Melanoms veröffentlicht. Im Vergleich zum Melanom, MCC und cuSCC ist die Datenlage im Basalzellkarzinom bisher noch sehr begrenzt, so dass diese Erkrankung hier nicht weiter besprochen wird.

Die Rationale für den möglichen zusätzlichen Vorteil einer neoadjuvanten Therapie ist bei allen Entitäten ähnlich: Sie könnte weniger ausgedehnte Eingriffe ermöglichen und die Lymphknotendissektion überflüssig machen, wodurch Morbidität und Disfiguration reduziert werden. Zudem scheint die neoadjuvante Therapie das rezidivfreie Überleben signifikant stärker zu verbessern als die adjuvante Therapie. Im Falle der ICI‐Therapie könnte dies auf eine verstärkte T‐Zell‐Expansion während der neoadjuvanten Phase zurückzuführen sein. Diese Expansion wird wahrscheinlich durch eine verbesserte Immunantwort auf die noch in situ vorhandenen Tumorzellen ausgelöst.^6^, ^7^ Ein zusätzlicher Vorteil der neoadjuvanten Therapie besteht in der frühzeitigen Beurteilung des Ansprechens, da durch die histopathologische Analyse des Resektionspräparats eine objektive Einschätzung der Wirksamkeit ermöglicht wird. Dies kann sowohl prognostische Informationen liefern als auch die weitere Therapieplanung unterstützen.

**ABBILDUNG 1:**
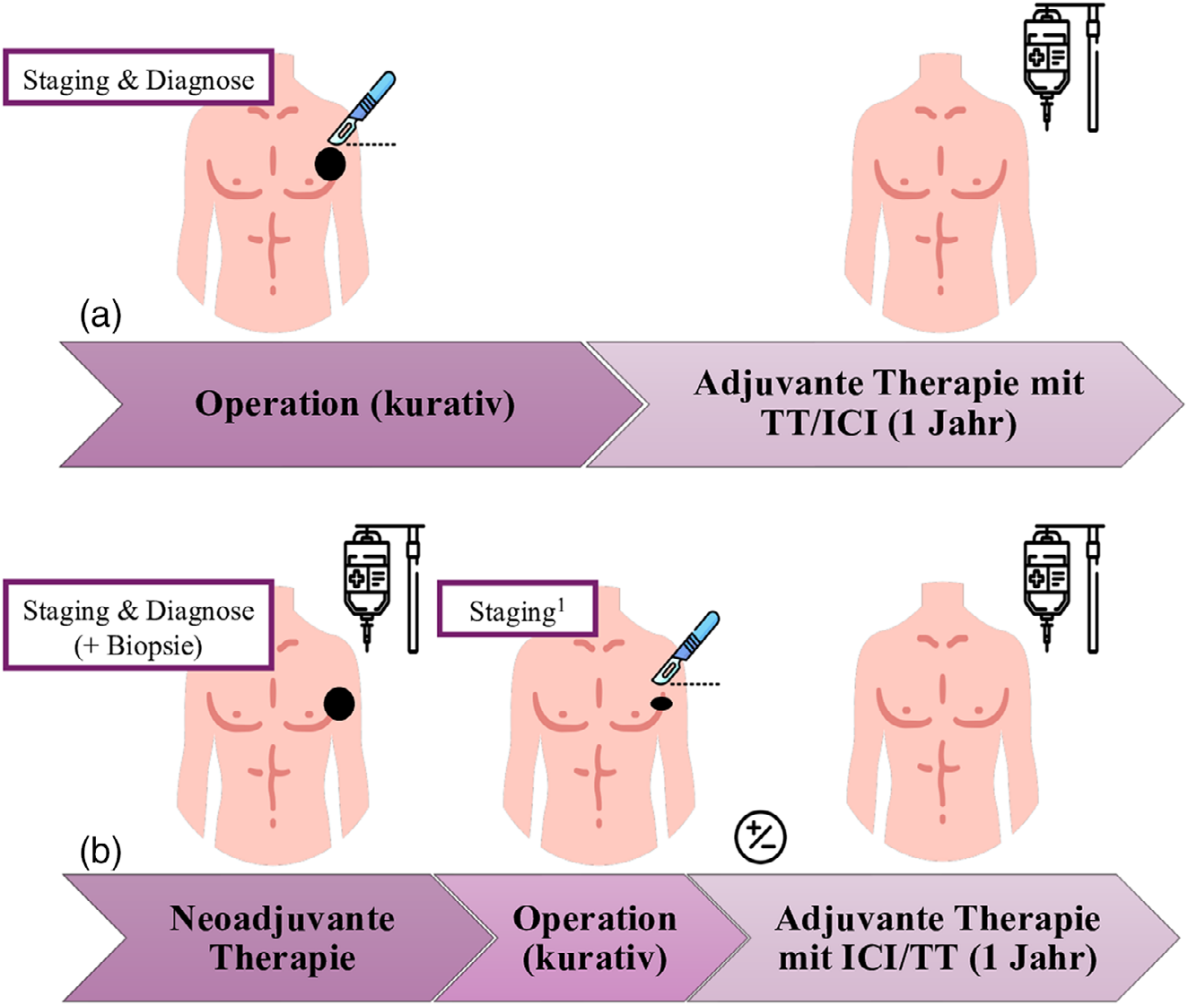
Gegenüberstellung der Konzepte (a) adjuvante versus (b) neoadjuvante plus gegebenenfalls adjuvante Therapie beim Melanom. In (b) ist die Möglichkeit des Verzichts auf eine adjuvante Therapie in Abhängigkeit vom pathologischen Ansprechen und vom Studienprotokoll dargestellt. *Abk*.: TT, gezielte Therapie; ICI, Immuncheckpoint‐Inhibitor. Icons von MaxIcons und Freepik, alle von flaticon.com. ^1^Präoperative Bildgebung zur Beurteilung des radiologischen Ansprechens, der Operabilität sowie zum weiteren Ausschluss einer Fernmetastasierung.

Die klinische Indikation für eine neoadjuvante Therapie bei malignen Hauttumoren kann bei lokoregionären oder oligometastatischen, aber operablen Erkrankungen (Stadium II–IV) im interdisziplinären Kontext erwogen werden. Darüber hinaus sollte die leitliniengerechte Standardtherapie angeboten werden, die – abhängig von der Tumorentität – in einer primären operativen Tumorresektion mit anschließender adjuvanter Behandlung besteht, etwa durch eine Radiotherapie und/oder eine systemische Therapie. Abbildung  1 veranschaulicht den Vergleich zwischen neoadjuvanten und rein adjuvanten Therapieregimen beim Melanom.

Das pathologische Ansprechen der neoadjuvanten Therapie wird standardmäßig postoperativ nach den Kriterien in Tabelle 1 bewertet.^8^ Hierauf basierend wird je nach Studien‐ und Therapieregime die mögliche adjuvante Therapie eingeleitet.

**TABELLE 1:** Darstellung der Kriterien des International Neoadjuvant Melanoma Consortium für das pathologische Ansprechen.

Pathologisches Ansprechen	Definition
pCR	Keine vitalen Tumorzellen
npCR/MPR	≤ 10 % vitale Tumorzellen
pPR	> 10–≤ 50 % vitale Tumorzellen
pNR	> 50 % vitale Tumorzellen

*Abk*.: npCR/MPR, nahezu pathologische Komplettremission/major pathological response; pCR, pathologische Komplettremission; pNR, pathologisches Nichtansprechen; pPR, pathologisches Teilansprechen

## NEOADJUVANTE THERAPIE DES KUTANEN PLATTENEPITHELKARZINOMS

Das cuSCC ist die zweithäufigste Hautkrebsform mit einer Inzidenz von jährlich 54 Neuerkrankungen bei Männern und 26 Neuerkrankungen bei Frauen pro 100 000 Einwohner in Deutschland.^9^ In den meisten Fällen ermöglicht eine operative Therapie die Heilung. Bei hohem Rezidivrisiko kann eine adjuvante Strahlentherapie erwogen werden. Etwa 2 %–5 % der Patienten entwickeln ein fortgeschrittenes, nichtresektables cuSCC (5‐Jahres‐Gesamtüberleben [OS] 30 %).^10^ Seit 2019 ist der PD‐1‐Inhibitor Cemiplimab in Europa für fortgeschrittene und metastasierte cuSCC zugelassen.^11^ Eine neoadjuvante Therapie ist für das cuSCC bislang nicht zugelassen.

## NEOADJUVANTE PD‐1‐INHIBITION

Nach einer vorangegangenen Pilotstudie wurde 2022 eine multizentrische Phase‐II‐Studie publiziert, welche die neoadjuvante Therapie mit dem PD‐1‐Inhibitor Cemiplimab bei fortgeschrittenen, aber operablen cuSCCs im Stadium II–IV (M0) untersuchte.^12^ Insgesamt erhielten 79 Patienten präoperativ vier Gaben Cemiplimab (jeweils 350 mg) in dreiwöchentlichen Abständen. Postoperativ erfolgte nach Ermessen des Behandlers entweder eine adjuvante Behandlung mit Cemiplimab über bis zu 48 Wochen, eine Strahlentherapie oder eine Nachbeobachtung. Die Ergebnisse zeigten ein komplettes pathologisches Ansprechen (pCR) bei 51 %, ein pathologisches Gesamtansprechen bei 64 % der Patienten. Nach 12 Monaten hatte keiner der Patienten mit pCR ein Rezidiv. In der Neoadjuvans traten Grad‐3‐Toxizitäten bei 4 % der Patienten auf.^13^

Aktuell läuft die Phase‐II‐Studie NEO‐CESQ, in der Patienten mit resektablem cuSCC im Stadium III/IV eine neoadjuvante und adjuvante Therapie mit Cemiplimab erhalten. Im neoadjuvanten Setting erfolgte die Gabe von Cemiplimab in zwei Zyklen à 350 mg im Abstand von jeweils 3 Wochen, gefolgt von einer postoperativen Therapie über 1 Jahr. Erste Ergebnisse aus dem Jahr 2023 zeigten ein pathologisches Gesamtansprechen bei 52 % (12/23) der Patienten ohne Grad‐3/4‐Toxizitäten.^14^

## NEOADJUVANTE KOMBINATIONSTHERAPIE PD‐1‐ PLUS CTLA‐4‐INHIBITION

Die niederländische Phase‐II‐Studie MATISSE vergleicht derzeit die neoadjuvante Gabe von Nivolumab mit einer Kombination aus Ipilimumab und Nivolumab im Stadium I–IVa. Vorläufige Daten zeigen ein nahezu komplettes pathologisches Ansprechen (major pathological response; MPR) von 40 % unter Nivolumab‐Monotherapie und 53 % unter Kombinationstherapie. Neun von 50 Patienten lehnten nach neoadjuvanter Therapie aufgrund einer klinischen Komplettremission, die im FDG‐PET‐CT bestätigt wurde, eine Operation oder Strahlentherapie ab. Nach 12 Monaten zeigte keiner dieser Patienten ein Rezidiv.^15^ Die Follow‐up‐Daten, insbesondere zu den Sicherheitsprofilen der beiden Therapiearme, stehen noch aus. Die MATISSE‐Studie deutet darauf hin, dass die neoadjuvante Anti‐PD‐1‐Therapie beim cuSCC in einigen Fällen kurativ wirken und ausgedehnte Operationen oder Bestrahlungen ersetzen könnte.

## NEOADJUVANTE MELANOMTHERAPIE

Im Jahr 2011 hat mit der Einführung von ICI und etwas später der BRAF/MEK‐Inhibitoren eine neue Ära für die Melanomtherapie begonnen. Das mediane OS für nichtresektable oder metastasierte Melanome beträgt nun 6,5 Jahre.^16^ Kürzlich wurde die Indikation für PD‐1‐Blocker auf die adjuvante Therapie im Stadium II ausgeweitet. Im Stadium III sind nach vollständiger Resektion die BRAF/MEK‐Inhibitoren Dabrafenib/Trametinib sowie die PD‐1‐Blocker Nivolumab (auch adjuvant im Stadium IV zugelassen) und Pembrolizumab zugelassen, da sie das Rezidivrisiko signifikant senken. Ein OS‐Vorteil durch adjuvante Systemtherapien konnte jedoch nicht nachgewiesen werden.^17^, ^18^, ^19^

## NEOADJUVANTE BRAF/MEK‐INHIBITION MIT DABRAFENIB PLUS TRAMETININB

Eine der ersten untersuchten neoadjuvanten Therapiestrategien beim Melanom war die Kombination von Dabrafenib/Trametinib. In einer 2018 von Amaria et al. veröffentlichten Phase‐II‐Studie wurden in zwei Behandlungsarmen Patienten im Stadium III–IV (M1a), die eine BRAFV600‐Mutation trugen, verglichen.^20^ Der erste Arm erhielt eine primäre Operation mit adjuvanter Standardtherapie nach Behandlerwahl. Der zweite Arm erhielt über 8 Wochen neoadjuvant Dabrafenib/Trametinib. Postoperativ folgte eine adjuvante Therapiephase über 44 Wochen. Die Studie wurde vorzeitig beendet, da die Interimsanalyse ein signifikant besseres ereignisfreies Überleben (EFS) der neoadjuvanten Therapie zeigte (19,7 vs. 2,9 Monate). Eine 2019 veröffentlichte einarmige Phase‐II‐Studie zeigte ein pathologisches Ansprechen von 100 % (17 pCR, 18 pathologische Teilremission [pPR]). Das 2‐Jahres‐RFS in Patienten mit pCR vs. non‐pCR war 63 % vs. 24 % und damit im indirekten Vergleich zu Immuntherapieschemata deutlich unterlegen.^21^ Die NeoTrio‐Studie untersucht die Kombination von BRAF/MEK und Pembrolizumab bei Stadium III‐BRAF‐mutierten Patienten. Vorläufige Daten zeigen eine bessere pCR bei simultaner Gabe im Vergleich zur sequenziellen Gabe oder Pembrolizumab‐Monotherapie, jedoch war die Toxizität bei simultaner versus sequenzieller Gabe deutlich höher (Grad‐3/4‐ Toxizität: 55 % vs. 25 %).^22^

## NEOADJUVANTE PD‐1‐INHIBITION MIT PEMBROLIZUMAB

In der ersten Studie zum neoadjuvanten Einsatz von Pembrolizumab zeigten 30 % der Patienten im Stadium III–IV nach nur einer Dosis eine pCR oder MPR. Alle Patienten mit pathologischem Ansprechen blieben nach einem medianen Follow‐up von 25 Monaten rezidivfrei.^23^

Mit der zweiarmigen SWOG1801‐Studie wurde 2023 die erste große Studie zur Neoadjuvans im resektablen Stadium III/IV publiziert.^24^ In dieser randomisierten Phase‐II‐Studie mit insgesamt 313 Patienten aus 90 US‐Zentren wurde die neoadjuvante plus adjuvante Pembrolizumab‐Therapie mit einer ausschließlich adjuvanten Pembrolizumab‐Therapie verglichen. In der experimentellen Gruppe erhielten die Patienten drei neoadjuvante Zyklen Pembrolizumab (200 mg in Abständen von 3 Wochen), gefolgt von einer Operation und 15 weiteren Infusionen Pembrolizumab in adjuvanter Intention. In der Vergleichsgruppe erfolgte die Operation sofort, gefolgt von 18 Infusionen Pembrolizumab in adjuvanter Intention – insgesamt genauso viele wie in der experimentellen Gruppe. Primärer Endpunkt war das EFS, wobei ein Ereignis als Rezidiv nach der Operation, Fortschreiten der bekannten Metastasierung, Toxizität, verspäteter Beginn der Adjuvans oder Tod definiert war. Nach einer medianen Nachbeobachtungszeit von 14,7 Monaten war die 2‐Jahres‐EFS‐Rate signifikant besser (72 % vs. 49 %). Die Toxizitätsrate war ähnlich (≥ Grad‐3‐Toxizitäten: 12 % vs. 14 %). Pathologisch hatten 21 % der Patienten eine pCR. Nach den RECIST‐Kriterien zeigte sich in der Bildgebung bei 6 % ein komplettes und bei 41 % ein partielles Ansprechen nach neoadjuvanter Therapie.

## IPILIMUMAB PLUS NIVOLUMAB

Jüngst wurden mehrere große Studien zur neoadjuvanten Kombination von Ipilimumab und Nivolumab veröffentlicht. In einer Phase‐II‐Studie von Amaria et al. erhielten 23 Patienten entweder vier Zyklen Nivolumab (3 mg/kg Körpergewicht [KG] in 2‐wöchigen Abständen) oder drei Zyklen einer Kombinationstherapie aus Ipilimumab (3 mg/kg KG) und Nivolumab (1 mg/kg KG) in 3‐wöchigen Abständen.^25^ Das radiologische Gesamtansprechen (ORR) nach RECIST lag bei 25 % vs. 73 %, die pCR‐Rate bei 25 % vs. 45 %. Nach einem medianen Follow‐up von 15,6 Monaten war das rezidivfreie Überleben (RFS) in der Kombinationsgruppe besser, allerdings traten signifikant häufiger Toxizitäten vom Grad ≥ 3 auf (73 % vs. 8 %). Zeitgleich erschien die OpACIN‐Studie, eine prospektive randomisierte Phase‐Ib/II‐Studie, in der Patienten mit makroskopischem Stadium‐III‐Melanom zweimal Ipilimumab (3 mg/kg KG) und Nivolumab (1 mg/kg KG) jeweils neoadjuvant und adjuvant erhielten.^6^ Vergleichend wurde in einem adjuvanten Arm dieselbe Kombinationstherapie viermal verabreicht. Bei 90 % der Patienten traten Toxizitäten vom Grad 3/4 bei klinisch hoher Wirksamkeit auf (pathologische Gesamtansprechrate: 78 %). Hieraufhin wurde in der Phase‐II‐Studie OpACIN‐neo eine optimierte Dosierung evaluiert.^26^ Das beste Verhältnis von Toxizität zu Wirksamkeit zeigte ein Flipped‐Dose‐Schema mit zwei Zyklen Ipilimumab (1 mg/kg KG) und Nivolumab (3 mg/kg KG) im Abstand von jeweils 3 Wochen. 47 % der Patienten erreichten eine pCR bei nur 20 % Grad 3/4‐Toxizitäten. Die 3‐Jahres‐Raten für RFS und OS der gesamten Kohorte betrugen 82 % bzw. 92 %. Bei pCR lag das RFS bei 95 %, bei pathologischem Nichtansprechen (pNR) bei 37 %.^27^ Eine später auch in anderen Studien bestätigte Diskrepanz zwischen radiologischem und pathologischem Ansprechen fiel durch höhere pathologische Ansprechraten auf.^28^ Ein pathologisches Ansprechen ist somit ein Biomarker, der den weiteren Verlauf der Melanomerkrankung zuverlässig vorhersagt.

Die OpACIN‐neo‐Daten wurden 2022 um die PRADO‐Erweiterungskohorte ergänzt, in der 99 Patienten die optimierte Kombinationstherapie erhielten. Die Korrelation zwischen dem pathologischen Ansprechen im Index‐Lymphknoten (ILN) und dem RFS wurde untersucht.^29^ Der ILN war definiert als die größte Lymphknotenmetastase zu Therapiebeginn, welche Clip‐markiert und nach zwei Zyklen entfernt wurde. Bei MPR/pCR wurde therapiedeeskalierend auf eine therapeutische Lymphknotendissektion (TLND) verzichtet, bei pPR erfolgte eine TLND, bei pNR zusätzlich eine adjuvante medikamentöse Therapie. Nach 24 Monaten lag das RFS bei MPR/pCR‐Patienten bei 93 %, was einen Verzicht auf TLND sicher erscheinen lässt. Weitere, randomisierte Studien sind jedoch nötig, um diese Therapiedeeskalation durchweg bei MPR/pCR im ILN zu empfehlen.

Mit der NADINA‐Studie wurde 2024 die größte und bisher einzige existierende Phase‐III‐Studie zur neoadjuvanten Therapie mit insgesamt 423 Patienten veröffentlicht und ein neuer Meilenstein in der Melanomtherapie erreicht.^30^ Eingeschlossen wurden Melanom‐Patienten im Stadium 3 mit mindestens einer resektablen Lymphknotenmetastase. Bis zu drei In‐transit‐Metastasen waren erlaubt, da seit 2020 mehrfach gezeigt wurde, dass auch bei diesen Patienten eine Neoadjuvans erfolgreich sein kann.^31^, ^32^ In Arm A erhielten Patienten neoadjuvant zweimal eine fixe Dosierung (flipped dose) Ipilimumab 80 mg plus Nivolumab 240 mg im Abstand von 3 Wochen, gefolgt von einer TLND. Bei MPR/pCR erfolgte keine adjuvante Therapie, ansonsten folgten zwölf Zyklen Nivolumab oder bei BRAFV600E/K‐Mutation Dabrafenib plus Trametinib (46 Wochen). Arm B erhielt eine Operation und anschließend zwölf Zyklen Nivolumab 480 mg q4w in adjuvanter Intention. Eine Radiotherapie war in beiden Armen außer bei MPR/pCR erlaubt. Primärer Endpunkt war das EFS (Progress zu nicht resektablem Melanom, Rezidiv oder Tod durch die Erkrankung/Behandlung). Nach 12 Monaten zeigte die neoadjuvante Gruppe ein signifikant besseres EFS (83,7 % vs. 57,2 %, p < 0,001). Bei 59 % der Patienten wurde ein MPR oder besser erreicht, sodass keine TLND oder adjuvante Systemtherapie erforderlich war. Das RFS lag bei 95,1 % (MPR) und 76 % (partielles pathologisches Ansprechen). Grad‐3/4‐Toxizitäten traten bei 29,7 % (neoadjuvant) und 14,7 % (adjuvant) der Patienten auf. Dies unterstreicht die Wirksamkeit der neoadjuvanten Therapie und das Ermöglichen einer Therapiedeeskalation.

## WEITERE SUBSTANZEN

### T‐VEC

Talimogene laherparepvec (T‐VEC) ist eine onkolytische Virustherapie, die auf dem Herpes‐Simplex‐Virus 1 basiert und intraläsional verabreicht wird. Sie ist für das nichtresektable Stadium III/IV (M1a) des Melanoms zugelassen. Eine Phase‐II‐Studie verglich die alleinige Operation mit neoadjuvantem T‐VEC. Der neoadjuvante Arm erreichte ein RFS von 29,5 % nach 2 Jahren, der operative Arm 16,5 %. Eine pCR wurde bei 17,1 % der Patienten in der neoadjuvanten Gruppe erreicht.^33^ Eine laufende Studie untersucht die neoadjuvante Kombination von T‐VEC und Nivolumab.^34^

### Daromun

Daromun ist eine intraläsionale Immuntherapie mit den Antikörper‐Cytokin‐Fusionen L19IL2 und L19TNF, wobei Interleukin (IL)‐2 und Tumornekrosefaktor (TNF)‐α gezielt in den Tumor abgegeben werden, was eine lokale Entzündung auslöst und die systemische Immunantwort verstärkt.^35^ L19 bewirkt dabei einen längeren Verbleib der Zytokine am Wirkort.^36^ Eine Phase‐II‐Studie zeigte Wirksamkeit bei Stadium IIIC‐IV, weshalb derzeit die PIVOTAL‐Phase‐III‐Studie zur neoadjuvanten Anwendung läuft. Hier wurde die neoadjuvante Therapie (13 Millionen IU L19IL2 und 400 µg L19TNF über 4 Wochen) mit einer Operation bei Patienten mit Haut‐ und/oder Lymphknoten Metastasen im resektablen Stadium III verglichen. Der neoadjuvante Arm erreichte in den vorläufigen Daten ein medianes RFS von 16,7 vs. 6,9 Monaten und eine pCR‐Rate von 21 %.^35^ Die Zulassung durch die *European Medicines Agency* (EMA) wurde auf Grundlage der Daten für das resektable Stadium IIIB–D beantragt, einschließlich vortherapierter Patienten.^37^ Somit könnte Daromun die erste zugelassene neoadjuvante Melanomtherapie werden.

### Pembrolizumab plus Vibostolimab oder Gebasaxturev

Eine 2025 publizierte Phase‐I/II‐Studie untersuchte die neoadjuvante Gabe von Pembrolizumab entweder in Kombination mit dem Anti‐TIGIT‐Antikörper Vibostolimab (T‐cell immunoreceptor with Ig and immunoreceptor tyrosine‐based inhibitory motif domains) oder mit dem onkolytischen Virus Gebasaxturev.^38^ TIGIT ist ein Rezeptor, der als Checkpoint eine Rolle in der Suppression der angeborenen und adaptiven Immunität spielt. Präklinisch zeigte Vibostolimab in Kombination mit PD‐1‐Inhibitoren eine Hemmung des Tumorwachstums. Gebasaxturev ist ein bioselektiertes, genetisch unmodifiziertes Coxsackievirus, welches nach Infektion direkt zu Tumorlyse führt und gleichzeitig die Tumorinfiltration durch CD8‐positive T‐Zellen fördert.^39^ In der Studie wurden drei neoadjuvante Arme verglichen: zweimal Pembrolizumab 200 mg plus Vibostolimab 200 mg im Abstand von 3 Wochen, versus einmal Pembrolizumab 400 mg plus bis zu fünf intraläsionale Injektionen mit Gebasaxturev gefolgt von einer Adjuvans mit achtmal Pembrolizumab 400 mg versus einmal Pembrolizumab 400 mg. Das pathologische Ansprechen lag jeweils bei 81 %, 52 % und 73 %, das EFS nach 18 Monaten bei 81 %, 72 % und 80 %. Grad‐3/4‐Toxizitätsraten lagen jeweils bei 15 %, 40 % und 27 %. Weder Vibostolimab noch Gebasaxturev sind weltweit zugelassen.

### Nivolumab plus Relatlimab

Die Kombination aus Nivolumab und dem Anti‐LAG3‐Antikörper Relatlimab erhielt 2022 die Zulassung für inoperables oder metastasiertes Melanom. Relatlimab ist ein humaner monoklonaler Antikörper, der LAG‐3 blockiert, somit LAG‐3‐vermittelte Immunsuppression reduziert und daher die Proliferation und Aktivierung von T‐Zellen fördert.^40^ Im Jahr 2022 wurde die erste Phase‐II‐Studie zur neoadjuvanten Therapie veröffentlicht, in der Patienten zweimal Nivolumab (480 mg) plus Relatlimab (160 mg) neoadjuvant alle 4 Wochen und anschließend zehn adjuvante Zyklen erhielten.^40^ Eine pCR wurde bei 57 % der Patienten erreicht, während 70 % ein pathologisches Gesamtansprechen zeigten. Nach 2 Jahren lag das RFS bei 92 % (Ansprechende) und 55 % (Nicht‐Ansprechende). Schwerwiegende Nebenwirkungen traten in der neoadjuvanten Phase nicht auf, 26 % entwickelten in der adjuvanten Phase eine Grad‐3/4‐Toxizität.

## NEOADJUVANTE THERAPIE IM MERKELZELLKARZINOM

Das Merkelzellkarzinom ist selten, aber in der Inzidenz zunehmend.^41^ In bis zu 80 % der Fälle ist das Merkelzell‐Polyomavirus integriert, alternativ beziehungsweise zusätzlich können Photokarzinogenesen zur Entstehung beitragen.^42^ Die Operation ist in frühen Stadien der Standard, jedoch mit hohen Rezidivraten und einer 5‐Jahres‐Überlebensrate von 50 %–60 % einhergehend.^43^ Das Merkelzellkarzinom spricht gut auf PD‐1/PD‐L1‐Checkpoint‐Inhibitoren an wie in einer einarmigen Phase‐II‐Studie gezeigt werden konnte, was 2017 zur europäischen Zulassung von Avelumab (PD‐L1‐Inhibitor) im Stadium IV führte (ORR: 33 %, OS: 12,6 Monate).^44^ In den USA ist Pembrolizumab nach positiven Ergebnissen einer Phase‐II‐Studie für das MCC im Stadium IV ebenfalls zugelassen (Ansprechrate 56 %).^45^ Aufgrund der hohen Rezidivraten in den Stadien‐III/IV untersuchte die Phase‐II‐ADMEC‐O‐Studie die adjuvante Nivolumab‐Therapie gegen Nachbeobachtung. Es zeigte sich eine absolute Rezidivrisikoreduktion von 9 %.^46^ Kürzlich wurden erste Daten zur neoadjuvanten Therapie des MCC veröffentlicht. Die Phase‐I/II‐CHECKMATE‐358‐Studie untersuchte neoadjuvant Nivolumab (zweimal 240 mg alle 2 Wochen) bei 39 Patienten mit resektablem MCC (Stadium IIA–IV), ohne festgelegte adjuvante Therapie.^47^ Diese konnte vom Behandelnden individuell eingesetzt werden. Die Ergebnisse waren vielversprechend: 47 % erreichten eine pCR, 15,4 % ein MPR, und 87,9 % eine radiologische Tumorreduktion. Ähnlich dem Melanom, bei dem 58 % der Patienten mit radiologischem SD pathologisch eine pCR oder pPR zeigten, war das pathologische Ansprechen oft besser als die Bildgebung.^28^ Nach 20 Monaten Follow‐up wurde das mediane RFS und OS nicht erreicht. Das RFS lag bei pCR‐Patienten signifikant höher (100 % vs. 59,6 % nach 12 Monaten).

## UNBEANTWORTETE FRAGEN UND ZUKUNFTSAUSSICHTEN

Die obigen Daten zeigen, dass eine neoadjuvante Therapie bei cuSCC, Melanom und MCC enormes Potenzial bietet, insbesondere in Bezug auf Verbesserung des EFS und RFS.

Die wahrscheinlichste Erklärung für den überlegenen Effekt der neoadjuvanten versus adjuvanten Therapie ist, dass präoperativ eine stärkere, diversere T‐Zell‐Antwort induziert wird, da der gesamte Tumor präsent ist und somit ein größeres Repertoire an T‐Zellen aktiviert wird.^30^ Die randomisierte Phase‐III‐Studie NADINA und gepoolte kleinere Studien zeigen, dass die Kombination von PD‐1‐Blockade mit CTLA‐4‐Inhibition die Wirksamkeit erhöht.^25^, ^48^, ^49^ Ipilimumab erweitert das tumorspezifische T‐Zell‐Repertoire und könnte so die antitumorale Immunantwort verbessern.^50^

Die obigen Studien zeigen zwar einen großen Nutzen der neoadjuvanten Therapie, es bleibt aber unklar, welche Patientengruppen am stärksten profitieren. Beim Melanom wurden nur wenige Patienten im Stadium IV mit resektablen Einzelmetastasen eingeschlossen, was die Relevanz der Daten in dieser Therapiesituation fraglich macht. Auch Patienten im Stadium III mit ausgedehnten Primärtumoren, Satelliten‐ oder In‐transit‐Metastasen ohne zusätzliche Lymphknotenbeteiligung waren unterrepräsentiert. Kleinere Studien zeigen jedoch ein vergleichbares Ansprechen auf die neoadjuvante Therapie bei synchronen Primärtumoren oder lokalen Hautmetastasen.^6^, ^28^, ^32^ Beim cuSCC ist zu beachten, dass sowohl in der Cemiplimab‐Studie von Gross et al. als auch in der MATISSE‐Studie viele Patienten mit T1–T2‐Stadium eingeschlossen wurden, was die EFS‐Daten verzerrt, da diese Patienten oft einen günstigen Verlauf zeigen. Zudem wurden in keiner der Studien immunsupprimierte Patienten eingeschlossen, die laut Leitlinie als Hochrisiko‐Patienten gelten.^51^ Der Ausschluss erfolgte aufgrund retrospektiver Daten, die auf eine geringere Wirksamkeit der ICI hindeuten bzw. aufgrund des Risikos, die Grunderkrankung oder eine Transplantatabstoßung durch die ICI zu triggern.^52^, ^53^, ^54^

Beim Melanom und cuSCC ist unklar, wer von einer adjuvanten Therapie nach Neoadjuvans profitiert. Die NADINA‐Studie zeigte, dass Melanom‐Patienten mit pCR oder MPR auch ohne adjuvante Therapie ein hervorragendes RFS haben. Die SWOG1801‐Studie ergab ähnlich gute RFS‐Daten bei Patienten, die beide Therapiephasen erhielten. Zur Therapieoptimierung wäre es wichtig zu klären, für welche Patienten die alleinige neoadjuvante Therapie mit dreimal Pembrolizumab ausreicht. Patienten mit pCR zeigen bei allen Hauttumoren das beste RFS, was durch eine gepoolte Melanom‐Analyse bestätigt wurde: Nach 2 Jahren lag das RFS bei 89 % für pCR/MPR‐Patienten vs. 50 % ohne pCR.^49^ Eine pCR könnte als Kriterium für eine Therapiedeeskalation fungieren. Unklar bleibt, ob bei MPR in einem Indexlymphknoten auf eine LAD verzichtet werden kann, wie in PRADO dargestellt.

Es bleibt fraglich, ob bei pNR eine Fortsetzung der Therapie sinnvoll ist, da eine „unwirksame“ Therapie weitergeführt würde. Das histologische Muster von pNR könnte als Diskriminator dienen.^55^ Nach der MATISSE‐Studie bleibt unklar, ob eine präoperative PD‐1‐Therapie beim cuSCC immer neoadjuvant ist, da individuell auch ein kurativer Ansatz ohne Operation eine Möglichkeit sein kann und somit der Begriff „neoadjuvant“ hinfällig wäre. Neun Patienten ohne Operation und adjuvante Therapie zeigten nach einem Jahr eine klinische Komplettremission und ein RFS von 100 %, was darauf hindeutet, dass eine Resektion nicht immer nötig ist. Für das MCC fehlen aussagekräftige Daten zur generellen Indikation einer adjuvanten Therapie, jedoch hatten Patienten ohne pathologisches Ansprechen nach neoadjuvanter Therapie in der zitierten Studie früh Rezidive, was die Notwendigkeit einer individuellen Adjuvans (auch nach Neoadjuvans) nahelegt.

Die Diskrepanz der Scores für das pathologische Ansprechen erschwert die Interpretation der Studiendaten. Frühere Studien definierten pCR als vollständiges Fehlen, pPR als ≤ 50 % und pNR als > 50 % vitale Tumorzellen. Neuere Studien führten die Begriffe „MPR“ beziehungsweise ncPR ein, definiert als 0–≤ 10 % vitale Tumorzellen, was die Vergleichbarkeit einschränkt. Erfreulich ist, dass das International Neoadjuvant Melanoma Consortium 2018 konsentierte Kategorien veröffentlichte, die in Studien und der klinischen Praxis verwendet werden sollten (Tabelle 1).^8^ Trotzdem braucht es eine klare Schulung der Patholog*innen der jeweiligen Zentren, um die Bewertung gemäß den Vorgaben durchzuführen, was im klinischen Alltag eine Hürde sein könnte.

Auch bei der neoadjuvanten Immuntherapie besteht das Problem der therapieassoziierten Nebenwirkungen. In den Studien zur neoadjuvanten Therapie bei Hautkrebs entwickelten 10 %–60 % der Patienten eine höhergradige immunvermittelte Nebenwirkung (Tabellen 2, 3). Toxizitäten können zu Verzögerungen der Operation und zu einem höheren Risiko für postoperative Komplikationen führen, besonders wenn Glukokortikosteroidtherapien notwendig sind. Die NADINA‐ und SWOG1801‐Studien zeigten jedoch positive Ergebnisse: In der NADINA‐Studie musste die Operation nur bei 1 % (n = 3) und in der SWOG‐Studie bei nur einem Teilnehmenden aufgrund von Toxizitäten abgesagt werden.^24^, ^30^ Dennoch kommt es zu OP‐Verzögerungen durch die neoadjuvante Therapie. Auch wenn nur wenige Patienten während der Therapie einen Progress erlebten (2 % in NADINA, 7,7 % in SWOG), sollten alle Patienten über das Risiko eines Tumorfortschreitens, das zur Inoperabilität führen könnte, aufgeklärt werden.^24^, ^30^

**TABELLE 2:** Vergleich neoadjuvanter Therapieschemata beim Plattenepithelkarzinom.

Studie	Behandlung (neoadjuvant)	n Pat.	pCR (%)	MPR (%)	AE G3–4 (%)
NCT04154943	4 x Cemiplimab 350 mg alle 3 Wochen	79	51	13	18
MATISSE	1 x IPI 1 mg/kg + NIVO 3 mg/kg gefolgt von 1 x NIVO 3 mg/kg alle 2 Wochen	50	NR	53	12
	2 x NIVO 3 mg/kg alle 2 Wochen		NR	40	
NEO‐CESQ	2 x Cemiplimab 350 mg im Abstand von 3 Wochen	23	39	8	0

*Abk*.: AE, Nebenwirkungen; IPI Ipilimumab; MPR, major pathologische Remission; n, Anzahl; NIVO, Nivolumab; NR, nicht berichtet; pCR, pathologische Komplettremission.

**TABELLE 3:** Vergleich neoadjuvanter Therapieschemata beim Melanom.

Studie	Behandlung (neoadjuvant)	n Pat.	pCR (%)	MPR (%)	AE G3–4 (%)
NCT02231775	Dabrafenib 150 mg + Trametinib 2 mg/Tag	13	58	NR	61
NeoCombi	12 Wochen Dabrafenib 150 mg + Trametinib 2 mg/Tag	35	49	NR	29
NCT02519322	2 x Nivolumab 480 mg + Relatlimab 160 mg alle 4 Wochen	30	57	7	26
Opacin	2 x IPI 3 mg/kg KG+ NIVO 1 mg/kg KG alle 3 Wochen	9	33	66	90
NCT02519322	NIVO 3 mg/kg KG alle 2 Wochen bis zu 4 x	12	25	NR	8
	3 x IPI 3mg/kg KG+ NIVO 1 mg/kg KG im Abstand von 3 Wochen	11	45		73
OpacinNeo	2 x IPI 3 mg/kg + NIVO 1 mg/kg KG im Abstand von 3 Wochen	30	47	70	40
	2 x IPI 1 mg/kg + NIVO 3 mg/kg KG im Abstand von 3 Wochen	30	57	64	20
	2 x IPI 3 mg/kg KG gefolgt von 2 x NIVO 3 mg/kg KG	26	23	46	50
PRADO	2 x IPI 1 mg/kg KG + NIVO 3 mg/kg KG im Abstand von 3 Wochen	99	49	61	22
*SWOG 1801*	3 x Pembrolizumab 200 mg im Abstand von 3 Wochen	154	21	NR	12
*NADINA*	2 x IPI 80mg + NIVO 240 mg im Abstand von 3 Wochen	212	47,2	59	29,7

*Abk*.: AE, Nebenwirkungen; IPI, Ipilimumab; MPR, major pathologische Remission; n, Anzahl; NIVO, Nivolumab; NR, nicht berichtet; pCR, pathologische Komplettremission

Kursiv hervorgehoben werden die beiden neusten und wichtigsten Studien

Mitunter das größte Hindernis im klinischen Alltag ist die fehlende Zulassung neoadjuvanter Melanomtherapien in Europa. Zusätzlich ist in Deutschland auch eine Erstattung nicht automatisch gegeben. In Australien, Italien und anderen Ländern wurde die Kostenübernahme für das SWOG1801‐Schema von den Behörden zugesagt. In Deutschland ist die Erstattung neoadjuvanter Therapien nach SWOG1801‐ oder NADINA‐Schema vorab mit den Kostenträgern zu klären. Aufgrund prospektiver, randomisierter Daten besteht eine valide Datenlage, auf welche verwiesen werden sollte. Bei Plattenepithel‐ und Merkelzellkarzinomen beschränkt sich die Datenlage auf einarmige Studien, sodass die Erstattungsbeantragung eine größere Hürde darstellt und weitere Studien zur Argumentationsfestigung benötigt werden.

Ein weiteres praktisches Problem im klinischen Alltag stellt die Terminkoordination dar. Für eine reibungslose neoadjuvante Versorgung ist eine enge Abstimmung von Infusionsterminen, Staginguntersuchungen und Operationsterminen unerlässlich.

Neben den bereits diskutierten neoadjuvanten Therapieansätzen bei Hautkrebs befinden sich weitere klinische Studien in der Rekrutierungs‐ oder Anlaufphase. Ein Beispiel hierfür ist die Phase‐II‐Studie IOB‐032, in der die Sicherheit und Wirksamkeit einer Kombination aus dem Peptidimpfstoff IO102‐IO103 und Pembrolizumab als neoadjuvante und adjuvante Therapie bei Patienten mit resezierbarem Melanom untersucht wird.^56^ Wie bereits erwähnt, befinden sich viele Studien noch in der Durchführung; umfassendere Auswertungen und Ergebnisse sind in naher Zukunft zu erwarten.

### FAZIT

Die neoadjuvante Therapie bei Hautkrebs, insbesondere beim Melanom, zeigt großes Potenzial, dennoch bleiben Fragen zur Patientenselektion und den optimalen Therapieschemata offen. Weitere Untersuchungen sind nötig, um die Therapieansätze zu verbessern und den größtmöglichen Nutzen für die Patienten zu erzielen. Bereits jetzt sollte aber eine neoadjuvante Therapie bei allen Melanompatienten mit klinisch detektierbaren Lymphknotenmetastasen im Stadium III empfohlen werden und im operablen Stadium IV diskutiert werden, da die vorliegenden Daten zeigen, dass sie im Vergleich zum bisherigen Therapiestandard den Verlauf der Erkrankung deutlich verbessert.

## DANKSAGUNG

Open access Veröffentlichung ermöglicht und organisiert durch Projekt DEAL.

## INTERESSENKONFLIKT

J.K. erhielt Honorare von Bristol‐Myers Squibb, Sanofi, SUN Pharma und Amgen für die Teilnahme an Advisory Boards oder für Referententätigkeiten sowie Reiseunterstützung von Pierre Fabre Pharma. B.S. erhielt Honorare von SUN Pharma, Immunocore, Bristol‐Myers Squibb, Sanofi, Novartis, Regeneron, Pierre Fabre Pharma und Blueprint Medicines für die Teilnahme an Advisory Boards oder für Referententätigkeiten. L.D. gibt an, dass kein Interessenkonflikt besteht.
